# Supplementary material for: The Stages and Grades of Periodontitis Are Risk Indicators for Peri-Implant Diseases—A Long-Term Retrospective Study
Source: J Pers Med. 2022 Oct 15;12(10):1723. doi: 10.3390/jpm12101723 (PMC9604891; doi:10.3390/jpm12101723)
Supplement: Supplementary file 1 [file jpm-12-01723-s001.zip › jpm-1882649-supplementary.pdf]

Journal of Personalized Medicine

MDPI

Table S1: 【**Patient level**】**Fisher's exact test**（**Treatment outcome** × **Stage**） P=0.020

Stage		PI（L）	PI	MBL≧3mm	MBL＜3mm	LoO	Total
I	Count	0.00	0.00	1.00	11.00	2.00	14.00
	Expected Count	0.67	1.17	1.17	10.17	0.83	14.00
	Adjusted Residual	-0.92	-1.24	-0.18	0.55	1.44	
II	Count	0.00	1.00	1.00	13.00	0.00	15.00
	Expected Count	0.71	1.25	1.25	10.89	0.89	15.00
	Adjusted Residual	-0.96	-0.26	-0.26	1.35	-1.08	
III	Count	0.00	3.00	1.00	27.00	3.00	34.00
	Expected Count	1.62	2.83	2.83	24.69	2.02	34.00
	Adjusted Residual	-1.69	0.13	-1.47	1.15	0.92	
IV	Count	4.00	3.00	4.00	10.00	0.00	21.00
	Expected Count	1.00	1.75	1.75	15.25	1.25	21.00
	Adjusted Residual	**3.55**	1.14	**2.05**	**-2.97**	-1.33	
Total	Count	4.00	7.00	7.00	61.00	5.00	84.00
	Expected Count	4.00	7.00	7.00	61.00	5.00	84.00

Table S2: 【**Patient level**】**Fisher's exact test**（**Treatment outcome** × Grade） P=0.307

Grade		PI（L）	PI	MBL≧3mm	MBL＜3mm	LoO	Total
A	Count	0.00	0.00	0.00	14.00	2.00	16.00
	Expected Count	0.76	1.33	1.33	11.62	0.95	16.00
	Adjusted Residual	-0.99	-1.34	-1.34	1.48	1.23	
B	Count	0.00	3.00	2.00	17.00	0.00	22.00
	Expected Count	1.05	1.83	1.83	15.98	1.31	22.00
	Adjusted Residual	-1.22	1.05	0.15	0.57	-1.37	
C	Count	4.00	4.00	5.00	30.00	3.00	46.00
	Expected Count	2.19	3.83	3.83	33.40	2.74	46.00
	Adjusted Residual	1.86	0.13	0.93	-1.67	0.24	
Total	Count	4.00	7.00	7.00	61.00	5.00	84.00
	Expected Count	4.00	7.00	7.00	61.00	5.00	84.00

Table S3: **【Implant level】Fisher's exact test（Treatment outcome × Stage）** P＝0.021

Stage		PI（L）	PI	MBL≧3mm	MBL＜3mm	LoO	Total
	**Count**	0.00	0.00	2.00	39.00	2.00	43.00
**I**	**Expected Count**	0.66	1.85	2.12	37.44	0.93	43.00
	**Adjusted Residual**	-0.88	-1.49	-0.09	0.76	1.21	
	**Count**	0.00	2.00	2.00	48.00	0.00	52.00
**Ⅱ**	**Expected Count**	0.80	2.24	2.56	45.28	1.12	52.00
	**Adjusted Residual**	-0.98	-0.18	-0.39	1.23	-1.17	
	**Count**	0.00	3.00	2.00	101.00	4.00	110.00
**Ⅲ**	**Expected Count**	1.69	4.74	5.42	95.78	2.37	110.00
	**Adjusted Residual**	-1.61	-1.00	-1.85	1.82	1.32	
	**Count**	5.00	9.00	10.00	95.00	1.00	120.00
**Ⅳ**	**Expected Count**	1.85	5.17	5.91	104.49	2.58	120.00
	**Adjusted Residual**	**2.95**	**2.17**	**2.17**	**-3.25**	-1.25	
**Total**	**Count**	5.00	14.00	16.00	283.00	7.00	325.00
	**Expected Count**	5.00	14.00	16.00	283.00	7.00	325.00

Table S4: **【Implant level】Fisher's exact test（Treatment outcome × Grade）** P=0.359

Grade		PI（L）	PI	MBL≧3mm	MBL＜3mm	LoO	Total
	**Count**	0.00	0.00	1.00	37.00	2.00	40.00
**A**	**Expected Count**	0.62	1.72	1.97	34.83	0.86	40.00
	**Adjusted Residual**	-0.84	-1.43	-0.76	1.09	1.32	
	**Count**	0.00	5.00	3.00	70.00	0.00	78.00
**B**	**Expected Count**	1.20	3.36	3.84	67.92	1.68	78.00
	**Adjusted Residual**	-1.27	1.05	-0.50	0.81	-1.50	
	**Count**	5.00	9.00	12.00	176.00	5.00	207.00
**C**	**Expected Count**	3.18	8.92	10.19	180.25	4.46	207.00
	**Adjusted Residual**	1.70	0.05	0.96	-1.46	0.43	
**Total**	**Count**	5.00	14.00	16.00	283.00	7.00	325.00
	**Expected Count**	5.00	14.00	16.00	283.00	7.00	325.00
